# The Patient's Point of View

**Published:** 1910-02-19

**Authors:** Eva Richmond

**Affiliations:** Rockhampton, Falfield, Glos.


### THE PATIENT'S POINT OF VIEW.

*To the Editor of* The Hospital.

Sir,—May I point out a printer's error in my letter on above subject which you kindly print in your issue of the 12th, as it rather spoils the point of the letter. As there printed, the first patient told his wife, after being used as an "object-lesson," that he had had "the doctors" round his bed. What he really boasted of was that he had had "*ten* doctors," all trying to find out what was wrong. He was amused as well as pleased, for one of the "doctors" hazarded the opinion that the complaint, which caused much swelling of the joint of the great toe, was gout, whereupon one of the others called him "a fool," to the amusement of the listening patient.

Yours faithfully,

Rockhampton, Falfield, Glos. Eva Richmond.

